# Supplementary figures and images for: Interpretable dimensionality reduction and classification of mass spectrometry imaging data in a visceral pain model via non-negative matrix factorization
Source: PLoS One. 2024 Oct 10;19(10):e0300526. doi: 10.1371/journal.pone.0300526 (PMC11466421; doi:10.1371/journal.pone.0300526)

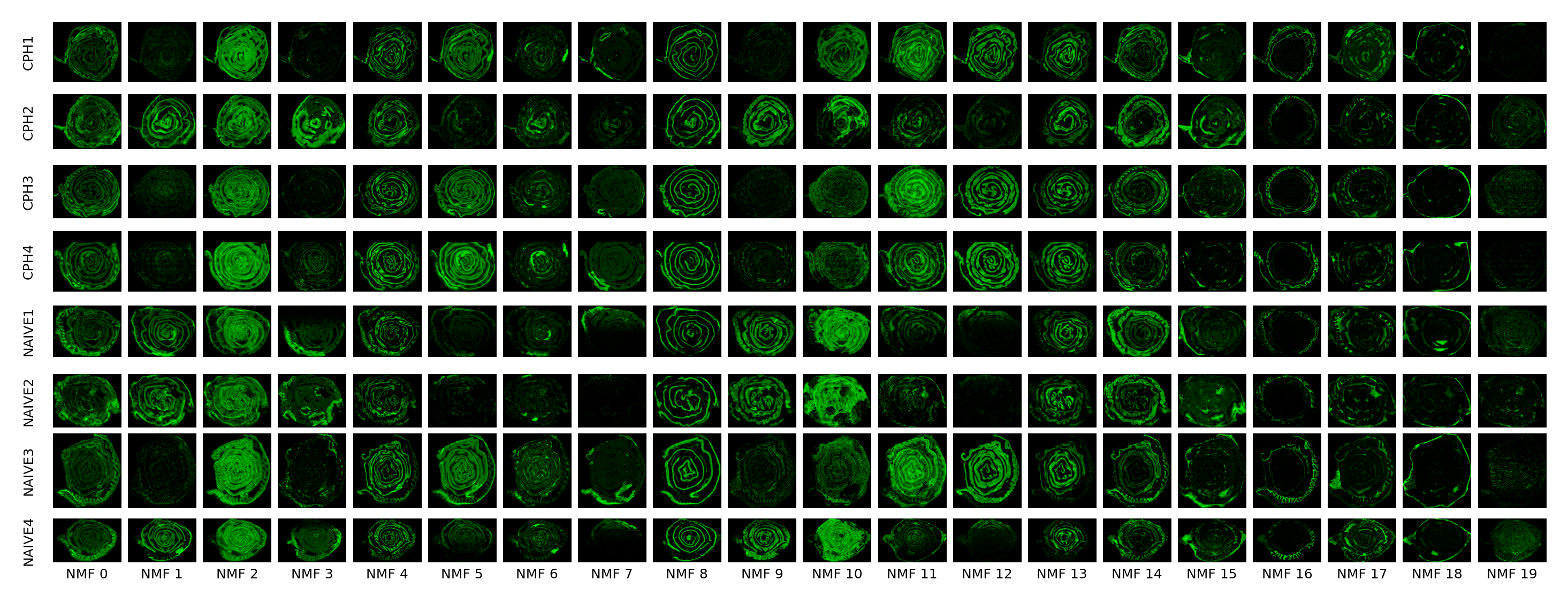

Supplement: S1 Fig — Each row shows 20 NMF spatial maps for each of the 8 datasets. The first four rows represent the CPH data and the last four rows represent the naïve data. (TIF) [file pone.0300526.s001.tif]

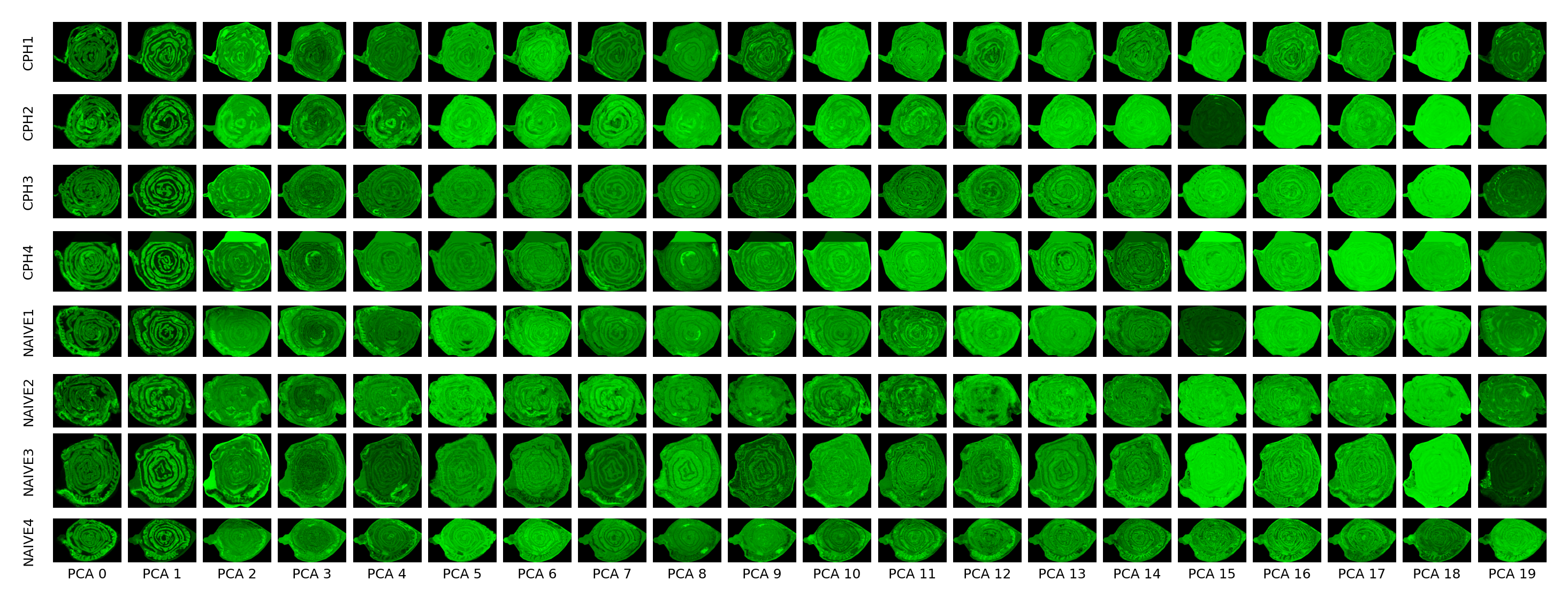

Supplement: S2 Fig — Each row shows 20 PCA spatial maps for each of the 8 datasets. The first four rows represent the CPH data and the last four rows represent the naïve data. (TIF) [file pone.0300526.s002.tif]

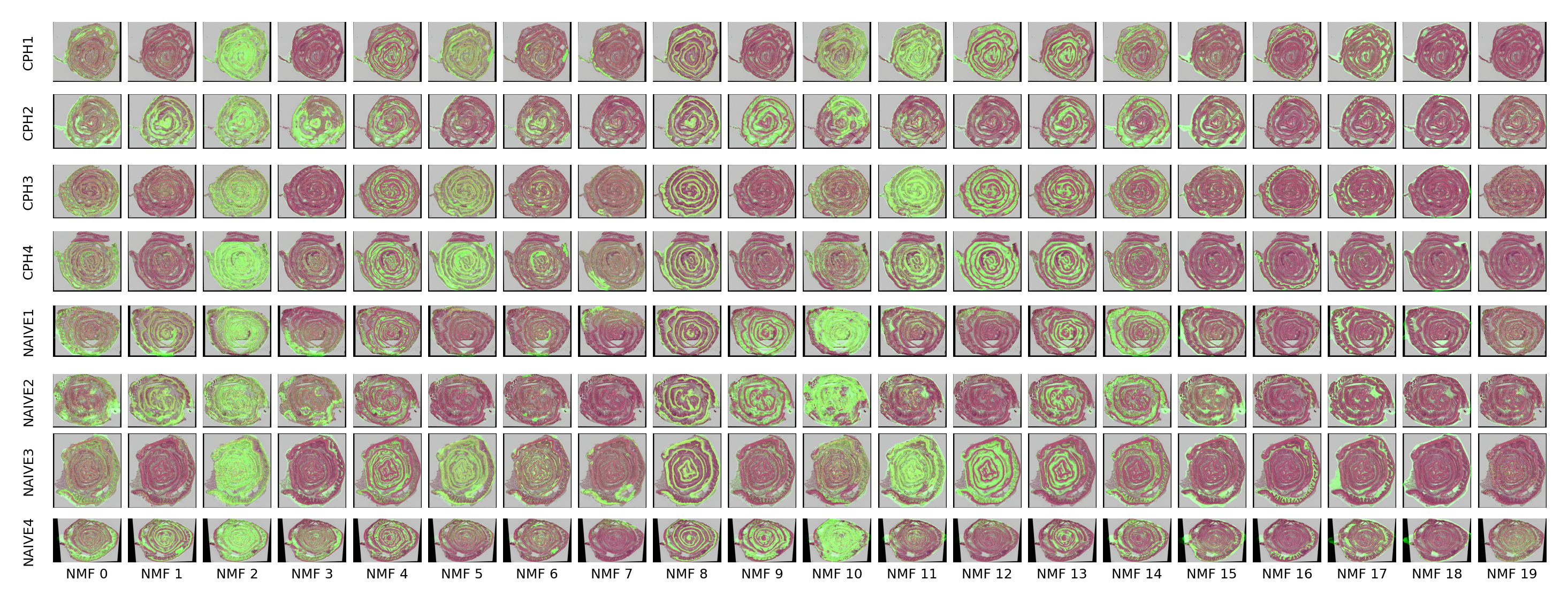

Supplement: S3 Fig — Overlay of 20 NMF spatial maps over the corresponding H&E image for each of the 8 datasets. The first four rows represent the CPH data and the last four rows represent the naïve data. (TIF) [file pone.0300526.s003.tif]
