# Supplementary material for: Interpretable dimensionality reduction and classification of mass spectrometry imaging data in a visceral pain model via non-negative matrix factorization
Source: PLoS One. 2024 Oct 10;19(10):e0300526. doi: 10.1371/journal.pone.0300526 (PMC11466421; doi:10.1371/journal.pone.0300526)
Supplement: Attachment — Submitted filename: Response_to_Reviewers_PONE-D-23-15506R1.docx [file pone.0300526.s004.docx]

**Response to the Reviewer Comments on**

**“Interpretable dimensionality reduction and classification of mass spectrometry imaging data in a visceral pain model via non-negative matrix factorization”**

**(PONE-D-23-15506R1)**

K. Pathirage^1,2,*^, A. Virmani^1,2,*^, A. J. Scott^3^, R. J. Traub^4^, R. K. Ernst^3^, R. Ghodssi^1,2^, B. Babadi^1,2^, and P. A. Abshire^1,2^

^1^ Institute for Systems Research, University of Maryland, College Park, Maryland, USA

^2^ Department of Electrical and Computer Engineering, University of Maryland, College Park, Maryland, USA

^3^ Department of Microbial Pathogenesis, University of Maryland, Baltimore, Maryland, USA

^4^ Department of Neural and Pain Sciences, University of Maryland School of Dentistry, Baltimore, Maryland, USA

We thank the reviewers for their thoughtful feedback on our manuscript. We have now substantially revised our manuscript by including new analyses, clarifying our results, and improving the clarity and rigor of our expositions. In summary, the major changes include:

**Preface**

1) Adding new analyses to justify the choice of the reduced dimensions (Fig. 4b) and extending the comparison of our proposed approach with existing methods (Fig. 10b and Table 1);
2) Substantially revising the abstract to emphasize the novel contributions of the paper and to be more accessible for a general audience;
3) Clarifying our main contributions in the context of existing recent work, in both the background and discussion sections, and highlighting the advantages, disadvantages, and limitations of our approach;
4) Substantially revising and enhancing the conclusion section; and
5) Improving the rigor and clarity of our presentation by significantly revising the text throughout the manuscript.

In what follows, we respond (in black) to the comments of the reviewers (in green) in a point-by-point fashion:

We have included an identical version of the manuscript that highlights all the major changes in blue for ease of tracking. We would like to thank the reviewing editor and the anonymous reviewers for their careful critique of our work and for their constructive and thorough feedback.

**Response to the Comments of Reviewer #1**

**Reviewer #1:**

Interpretable dimensionality reduction and classification of mass spectrometry imaging data in a visceral pain model via non-negative matrix factorization

The authors tried to analyze the Mass spectrometry imaging (MSI) data using non-negative matrix factorization. NMF used to reduce dimensionality and encountering spatial components.

In my opinion, this manuscript has these good points:

- The subject is interesting and might absorb many readers in the field;

- Nice written and well presenting the idea;

- Suitable analytical representations;

**Response:** We thank the reviewer for summarizing the strengths of our contributions.

Also, there are some suggestions that would increase the strength of the paper which is listed bellows;

- One of my major points about your article concerns the novelty of this article, authors should improve their novelty more highlighted. NMF is used for dimensionality reduction and spectroscopy data analysis (please google this to find more published contributions in the NMF for Hyperspectral, and spectroscopy), what is new in your article. Please specify your contributions.

**Response:** We thank the reviewer for raising this point on clarifying the novelty of our work. We have now revised our abstract to clarify the main contributions of the work as being three novel MSI data analysis techniques that leverage the spatial and spectral interpretability of NMF compressed MSI data (pp. 1 - 2).

We also added a section highlighting the connection to existing work in MSI data analysis to highlight the novel aspects of this work (pp. 5 - 6).

- Why did the size of data shrink from 100K to 20? There should a gap statistic similar approach to justify this.

**Response:**

We are not performing a traditional clustering, so gap statistics are not readily available or well defined for this approach. Instead we set a target error rate of 20% in reconstruction accuracy and used this criterion to select the number of components to retain in subsequent analysis. We have now revised Fig 4 to clearly show the selection criterion for the reduced dimension (for both PCA and NMF) (pp. 13)

- Similarity to clustering is also needed to the be highlighted and how does this manifest itself into the analysis.

**Response:** One of the key findings in this work is that NMF inherently produces components with spatially distinct structure obviating the need for an additional explicit clustering step. We have now added this explanation to the section on review of existing MSI data processing methods (pp. 5 - 6).

**Response to the Comments of Reviewer #2**

**Reviewer #2:**

In the presentation of the work, the article has a nice beginning; nonetheless, it has to be examined in order to help the reader comprehend “Interpretable dimensionality reduction and classification of mass spectrometry imaging data in a visceral pain model via non-negative matrix factorization." Despite the fact that I believe the work does not satisfy the requirements for publication in PLOS ONE and that there are some concerns that need to be addressed, I strongly propose a comprehensive review that will add value to the results that were acquired through discussion. The writers need to make some changes to the paper. On the other hand, I would like to provide the authors with the following remarks and suggestions:

**Response:** We thank the reviewer for their careful critique of our work and for providing a number of insightful suggestions to improve it.

1. The abstract does not communicate well; it must be revised.

**Response:** We have now revised our abstract to clarify the main contributions of the work as being three novel MSI data analysis techniques that leverage the spatial and spectral interpretability of NMF compressed MSI data (pp. 1 - 2).

2. In particular, it is not entirely evident how this publication contributes to the body of previous research when compared to other papers that have been published. Because of this, unable to propose that the current version be accepted.

**Response:** As noted above, we have now revised the abstract to better communicate the novelty of our work (pp. 1 - 2).

We have also added a more thorough literature review, specifically highlighting the novelty of our work in terms of NMF as it applies to downstream MSI data processing (pp. 5 - 6).

3. In Background and related work section, the author can introduce more literature and analyze its shortcomings to highlight the advantages and innovations of this paper.

**Response:** We have now added a more thorough literature review, specifically highlighting the novelty of our work on NMF data compression as it applies to downstream MSI data processing. We have also emphasized how our work relates to existing approaches (pp. 5 - 6).

4. The results themselves need to be explained, which is why there must be a section or paragraph dedicated to the discussion along with an appropriate comparison table of the suggested work.

**Response:** We have added subheadings in the discussion section to highlight the material related to results for distinct topics in our paper and improve readability. (pp. 22- 25)

Since the novelty of our work lies in data processing methodologies (i.e., how to integrate NMF into data processing pipelines for downstream analyses), it is hard to perform a quantitative comparison with other methods.

To establish a benchmark for how NMF and PCA perform on the novel dataset used in this work, we have added a table illustrating the comparison of PCA and NMF results on this novel dataset (pp. 22).

We have also modified Fig 10 to show how the methodologies described in this paper, when applied to NMF compressed MSI data, compare against MSI data compressed with PCA. (pp. 21)

5. More specifically, only simulated results were presented, and there was no attempt made to verify the suggested work by practical means in the tabular form.

**Response:** All of the results in this paper are based on a novel MSI dataset obtained from an animal model of comorbid visceral pain hypersensitivity, which is described in the Animal Model, Tissue Preparation, Mass spectrometry imaging and staining, and Datasets sections (pp. 7-9), We have reworded the abstract to emphasize the novelty of this dataset that we use for validating the methods (pp. 1 - 2). As noted above, we have also added a table comparing PCA and NMF approaches as applied to this novel dataset (pp. 22).

6. Add some recently proposed techniques (2020-2023) in the related work section of the manuscript.

**Response:** We have added a section on MSI data processing highlighting recent contributions and their connection to this work. We have updated the bibliography with the respective literature (references 21 - 24) (pp. 5 - 6).

7. There are too many spelling and grammar mistakes in the paper. It needs proper spelling and grammar checking.

**Response:** We have thoroughly proofread the manuscript for spelling and grammatical mistakes and have resolved all issues that we found.

8. Conclusion section should be extended by mentioning the advantages, disadvantages, and limitations of the study.

**Response:** We have significantly expanded the conclusion, describing in detail the advantages, disadvantages and limitations of our study (pp. 25 - 26).
